# Editorial: Supervision, control and learning for intelligent robot systems

**DOI:** 10.3389/frobt.2022.1050237

**Published:** 2022-10-12

**Authors:** Pedro U. Lima, Luca Iocchi

**Affiliations:** ^1^ Laboratory for Robotics and Engineering Systems (LARSyS), Instituto Superior Técnico (ISR), Lisbon, Portugal; ^2^ Department of Computer, Control and Management Engineering, Sapienza University of Rome, Rome, Italy

**Keywords:** robot control, artificial intelligence, reinforcement learning, formal methods, task planning

In recent decades, the path has been paved for complementary approaches to the supervision and control of robot systems blending automatic control, computer science, and artificial intelligence. The resulting methods are not limited to execute actions at a low level of abstraction, such as controlling the robot actuators and safely navigating in indoor/outdoor environments, further addressing higher-level decision-making, such as allocating tasks in a team of multi-robot systems, and then planning the task and executing the resulting plan.

This novel paradigm has been successfully achieved through different means, such as approaching the task planning and execution problems using discrete-event systems (DES), or combining DES with continuous state-space time-driven systems in hybrid systems, to solve task and motion planning problems. Logic-based specifications for such planners have been introduced, using different classes of temporal logic (TL). And, even more recently, the control community is using machine learning (ML), either exploiting reinforcement learning (RL) methods or revisiting the use of neural nets for nonlinear control.

The goal of this Research Topic is to display recent work surrounding supervision, control and learning robot tasks, blending automatic control and artificial intelligence approaches.

Four papers were accepted for the Research Topic.

“*Social Drone Sharing to Increase UAV Patrolling Autonomy in Pre- and Post-Emergency Scenarios*,” by Bisio et al., addresses the problem of coordinating teams of autonomous drones performing tasks in an area where social interactions with people will help in improving autonomy and general effectiveness of the missions. The article presents a cloud-based architecture for social drone missions, algorithms to optimize task performance, and experimental results assessing the performance of the proposed solution.

In “*Learning state-variable relationships in POMCP: a framework for mobile robots*”, Zuccotto et al. address the improvement of performance of planning under uncertainty by determining correlations between hidden state variables of a Partially Observable Markov Decision Process (POMDP). The proposed method exploits this correlation by transposing the information acquired by observing some state variables to their correlated unobserved state variables. The POMDP is implemented as Partially Observable Monte Carlo Planning (POMCP) and the acquired knowledge is represented with a Markov Random Field (MRF). The results of realistic simulations in two domains show improvements of online adapted MRF and POMCP with respect to the non-adapted version. The paper also introduces a ROS-based architecture that enables running the proposed method in real robot systems.

“*Task Roadmaps: Speeding Up Task Replanning*,” by Lager et al. focusses on optimal and efficient robot task replanning, by using Task Roadmaps that contains information to make replanning very efficient. The proposed method outperforms Mixed Integer Linear Programming (MILP) solvers and Planning Domain Definition Language (PDDL) planners in an interesting use case where a mobile manipulator is involved in delivery tasks in a warehouse scenario.

Using Deep Reinforcement Learning (DRL) to learn general tasks end-to-end is currently a very trendy Research Topic. In “*Network Layer Analysis for a RL-Based Robotic Reaching Task*,” Feldotto et al. propose methods to speed up the learning of the neural networks used in DRL, while keeping their performance levels, as well as to re-use them as pre-trained networks in similar tasks performed by robot manipulators with similar kinematics but different number of joints. To reach this goal, the authors introduce metrics that evaluate individual neuron activation and enable comparing those neurons activity with other neurons in the network. These metrics are used to reduce the redundancy and size of the neural network without significantly reducing their performance.

